# Development of Medical Imaging Data Standardization for Imaging-Based Observational Research: OMOP Common Data Model Extension

**DOI:** 10.1007/s10278-024-00982-6

**Published:** 2024-02-05

**Authors:** Woo Yeon Park, Kyulee Jeon, Teri Sippel Schmidt, Haridimos Kondylakis, Tarik Alkasab, Blake E. Dewey, Seng Chan You, Paul Nagy

**Affiliations:** 1 Biomedical Informatics and Data Science, Johns Hopkins University, 855 N Wolfe St, Rangos 616, Baltimore, MD USA; 2 Department of Biomedical Systems Informatics, Yonsei University College of Medicine, Seoul, Korea; 3 Institute for Innovation in Digital Healthcare, Yonsei University, Seoul, Korea; 4 Institute of Computer Science, Foundation of Research & Technology-Hellas (FORTH), Heraklion, Greece; 5 Department of Radiology, Massachusetts General Hospital, Boston, MA USA; 6 Department of Neurology, Johns Hopkins University, Baltimore, MD USA

**Keywords:** Data collection [MeSH], Data standardization, Observational research, Data integration, Multimodal data analysis

## Abstract

The rapid growth of artificial intelligence (AI) and deep learning techniques require access to large inter-institutional cohorts of data to enable the development of robust models, e.g., targeting the identification of disease biomarkers and quantifying disease progression and treatment efficacy. The Observational Medical Outcomes Partnership Common Data Model (OMOP CDM) has been designed to accommodate a harmonized representation of observational healthcare data. This study proposes the Medical Imaging CDM (MI-CDM) extension, adding two new tables and two vocabularies to the OMOP CDM to address the structural and semantic requirements to support imaging research. The tables provide the capabilities of linking DICOM data sources as well as tracking the provenance of imaging features derived from those images. The implementation of the extension enables phenotype definitions using imaging features and expanding standardized computable imaging biomarkers. This proposal offers a comprehensive and unified approach for conducting imaging research and outcome studies utilizing imaging features.

## Introduction

Observational health research leverages real-world clinical data extracted from electronic health records (EHR) and claims data sources. The most common data comes from structured coded fields, which only represents a small part of the electronic record. Clinical practice utilizes multiple data sources such as unstructured clinical notes and medical imaging to make clinical decisions. Increasing need to incorporate multimodal data, such as notes, waveforms, and images, as inputs in the machine learning methods is highlighted by recent studies [1–3]. The rich information captured in multimodal data increases prediction performance. The impact of integrating imaging and clinical variables consistently has demonstrated increased accuracy and area under the receiver operating characteristic curve (AUROC) over single modality analysis [2]. The limitations of current literature are collecting and harmonizing multimodal data in a standardized fashion [3]. Our proposal is to integrate clinical data stored in EHR with pixel-based imaging signatures in a standardized data structure and semantics to facilitate next generation outcome research.

The Observational Medical Outcomes Partnership Common Data Model (OMOP CDM) was created by the Observational Health Data Science and Informatics (OHDSI) community to represent structured observational healthcare data from electronic health records and claim reimbursement data [4]. As of 2022, structured data of 928 million patients have been harmonized into OMOP CDM from EHR, registries, and administrative claims [5]. The federated data network conducts multi-site studies without sharing patient-level data, but rather has code run locally on each dataset individually. Historically, the presence of imaging examinations has been identified in the OMOP common data model only as imaging procedure codes in the Procedure_occurrence table.

The medical image pixel-level data, scanner protocol, and patient information are formatted using Digital Imaging and Communication in Medicine (DICOM) and stored in Picture Archive and Communication Systems (PACS) as part of the medical-legal record. DICOM is the ubiquitous international standard for medical imaging. The DICOM standard storage format is designed to preserve storage disk space where pixel and metadata information is binary run length encoded. EHR data is defined in the HL7 standard, which promotes formats for human readability. The differing formats between EHR and imaging data have led to siloing research across these data sources. Researchers using EHR data often have access to the disease burden or patient outcomes common in medical records, while imaging researchers can study biomarkers and granular changes in diseases that are provided by medical imaging. Combining these sources will enable more holistic reproducible research. Our aim is to link algorithmically generated imaging measurements into the OMOP data model to harness these deeper phenotypes with the outcome measures tracked in the EHR.

Park et al. (2022) developed the first OMOP imaging extension for radiological imaging studies (R-CDM) [6]. While the R-CDM adeptly bridges structured data from OMOP CDM to DICOM headers, it does not encompass feature information pertinent to medical imaging and remains confined to radiological data. To address this gap, this study presents two novel tables and vocabularies for the OMOP CDM, fulfilling both structural and semantic needs for imaging research. The newly proposed tables not only enable the linkage of DICOM datasets but also trace the origin of imaging features extracted from the images. Furthermore, the revamped model incorporates medical images from diverse specialties like pathology, cardiology, and ophthalmology, ensuring provenance for enhanced reproducibility.

## Methods

The Medical Imaging Working Group (MI WG) for the OHDSI community was formed in 2021, comprised of imaging research scientists and observational health researchers familiar with OMOP CDM. The working group evaluated standard vocabularies, defined fields containing key imaging events, and identified limitations of the model. The working group started with the R-CDM in the development of the medical imaging extension [6]. Imaging researchers across the field were consulted to gather requirements and gain insights into the structure and usability of the proposed model. The principal clinical use case focused on longitudinal tracking of multiple lung nodules. Important attributes included CT acquisition parameters, nodule diameter, location, density, shape, and other phenotypes. A prototype using CT lung nodules was developed and demonstrated at the 2023 Society of Imaging Informatics in Medicine (SIIM) conference Hackathon.

The medical imaging extension proposal represents imaging characteristics through image occurrence and features tables. The tables developed are concordant with the OMOP CDM conventions. The semantics and structure of the proposed imaging tables are summarized in Table 1. Semantics define terminology and structure defines the arrangement of data [7].

**Table 1:** The framework of the proposed medical image standardized data model

	**Image_occurrence**	**Image_feature**
**Semantics**	DICOM – Properties of image acquisition such as function and technique SNOMED – Anatomical location, procedures, and diagnostic imaging modality	RadLex – Radiological findings that were found absent from or more specific than SNOMED SNOMED - Anatomical location LOINC - Measurements
**Structure**	1. Link to the DICOM images at the study or series level 2. Link Procedure_occurrence to Image_occurrence table 3. Provide provenance for Image_feature 4. Incorporate basic acquisition parameters into cohort definitions	1. Provide provenance from a clinical data table entry of a feature extracted from a medical image 2. Link to Image_occurrence to point to which images were used to create the feature at the study or series level. 3. Provide a method to group multiple related imaging features 4. Provide provenance of the algorithms and parameters used to create the Image_feature

## Proposed Medical Image Data Model

### OMOP CDM Medical Imaging Extension

**Fig. 1:**
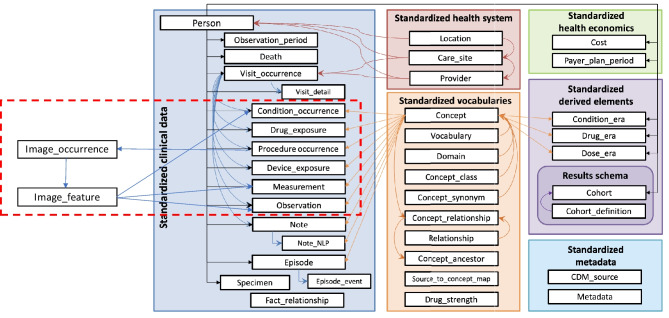
Incorporation of proposed medical image data model to existing OMOP CDM v5.4

The medical imaging extension follows conventions of the OMOP CDM. The CDM has a set of conventions encompassing concepts, structures, relationships, and other critical components of the data model design [8]. This study proposes adding two new tables to the CDM as shown in Fig. 1.

The medical imaging tables adhere to the OHDSI common data model conventions [4]. A table should be limited to a specific domain and link to the existing clinical data model with foreign keys to minimize duplication as shown in Figure 1. Related information, such as procedures and visits, can be extracted from the Procedure_occurrence and Visit_occurrence table, respectively. The new tables are linked to the Person table via person_id and datetime, following the OMOP CDM convention of patient-centric data model. This redundant foreign key convention simplifies analysis by allowing researchers to analyze the database table without the need of joining to the person table. The Image_feature table contains findings derived from the imaging study on the series level. Features will reside in clinical domain tables allowing existing OHDSI applications to incorporate imaging features in analysis. This will enable imaging researchers to combine imaging characteristics with other EHR features when they define cohorts from imaging findings. The fields of the tables follow the OMOP CDM conventions. Each row of the Image_feature table will contain a concept_id and a type_concept_id. The concept_id is the code for the vocabulary feature being measured. The type_concept_id describes the provenance of the source that feature came from.

The medical imaging domain leverages existing standard vocabularies, Systematized Nomenclature of Medicine – Clinical Terms (SNOMED CT) and Logical Observation Identifiers, Names and Codes (LOINC), and proposes adding specialized vocabularies to describe imaging events and finding, DICOM and Radiology Lexicon (RadLex).

## Results

### Standardization of Medical Imaging Data Semantics

The OMOP CDM standardizes discrete code systems, including LOINC and SNOMED CT, by using internal concept identifiers (concept_ids) to maintain a mapping between these codes systems. As part of this project, we propose extending the OMOP standard vocabularies to include mapping RadLex codes and DICOM value sets to OMOP concept_ids.

Every image is a “DICOM object” consisting of a header and pixel data and is identified by a globally unique identifier (UID). The header consists of well-defined attributes (also called “tags”) which clearly identify various pieces of information from the modality to acquisition parameters, such as kVp, to patient information, such as sex and age. DICOM includes domain-specific objects for radiology, cardiology, pathology, ophthalmology, and dentistry and is supported by nearly every medical imaging device.

DICOM attributes and Value Sets are comprised of chapters called “Parts.” The DICOM data model (patient-study-series-instance) and information objects (e.g., CT and MR IODs) are defined in DICOM Part 3 [9]. The DICOM standard has two types of data definitions: “attribute number” as the key and “Value Set” as the value. The first is called the “attribute number,” also called a “tag,” and is defined in DICOM Part 6 and consists of a “Group” and “Element,” written as (gggg,eeee) [10]. For example, “Modality” is (0008,0060). If DICOM has a defined Value Set for that attribute, the value is described in DICOM Part 16 “Context Groups” [11]. For example, enumerated values for Modality are MR, CT, NM, PT (PET), ES (Endoscopy), SM (Slide Microscopy), etc. In OMOP, both the variable name (DICOM attribute name) and the enumerated values (Value Sets) are added as concepts to the OMOP concept vocabulary. DICOM value sets can refer to SNOMED and LOINC.

RadLex is an addendum of the SNOMED CT vocabulary to include imaging findings used by the radiologist [12]. For example, the terms “ground glass” and “lobular” are often used to describe lung nodules. It should also be noted that there is an ACR and RSNA joint initiative to create radiology “Radlex Common Data Elements” (CDEs), which will play a role in coding the “key-value” pair of radiology imaging findings [13]. Other code systems may also be applicable for pathology and visible light images, although many of these codes are already defined in DICOM Part 16 in the appropriate Context Groups [11].

### Proposed Medical Image Extension Model

We have developed two new tables to be added to the OMOP CDM: Image_occurrence (Table 2) and Image_feature (Table 3). This extension follows the same structure conventions used to integrate source clinical notes and the provenance of natural language processing algorithms into the OMOP clinical domains [14].

**Table 2:** Image_occurrence table

Field	Required	Data type		Description
image_occurrence_id (PK)	Yes	integer	The unique key is given to an imaging study record (often referred to as the accession number or imaging order number)	
person_id (FK)	Yes	integer	The person_id of the Person for whom the procedure is recorded. This can be a system-generated code or adopted from original source	
procedure_occurrence_id (FK)	Yes	integer	The unique key is given to a procedure record for a person. Link to the Procedure_occurrence table	
visit_occurrence_id (FK)	No	integer	The unique key is given to the visit record for a person. Link to the Visit_occurrence table	
anatomic_site_concept_id (FK)	No	integer	Anatomical location of the imaging procedure by the medical acquisition device (gross anatomy). It maps the ANATOMIC_SITE_SOURCE_VALUE to a Standard Concept in the Spec Anatomic Site domain. This should be coded at the lowest level of granularity	
wadors_uri	No	varchar (max)	A Web Access to DICOM Objects via Restful Web Services Uniform Resource Identifier on study level.	
local_path	No	varchar (max)	Universal Naming Convention (UNC) path to the folder containing the image object file access via a storage block access protocol. (e.g., \\Server\Directory)	
image_occurrence_date	Yes	date	The date the imaging procedure occurred	
image_study_UID	Yes	varchar (250)	DICOM Study UID	
image_series_UID	Yes	varchar (250)	DICOM Series UID	
modality_concept_id	Yes	integer	The concept_id of DICOM-defined value (e.g., US, CT, MR, PT, DR, CR, NM)

**Table 3:** Image_feature table

Field	Required	Data type	Description
image_feature_id (PK)	Yes	integer	The unique key is given to an imaging feature
person_id (FK)	Yes	integer	The person_id of the Person table for whom the procedure is recorded. This can be a system-generated code or adopted from original source
image_occurrence_id (FK)	Yes	integer	The unique key of the Image_occurrence table
image_feature_event_field_concept_id (FK)	No	integer	The concept_id of the domain table that feature is stored in Measurement, Observation, etc. This concept should be used with the image_feature_event_id. The foreign key links to the Concept table
image_feature_event_id	No	integer	The primary key id of the domain table (e.g., Measurement) that feature is stored
image_feature_concept_id	Yes	integer	Concept_id of standard vocabulary—often a LOINC or RadLex of image features
image_feature_type_concept_id	Yes	integer	This field can be used to determine the provenance of the imaging features (e.g., DICOM SR, algorithms used on images)
image_finding_concept_id	No	integer	RadLex or other terms of the groupings of image feature (e.g., nodule)
image_finding_id	No	integer	Integer for linking related image features. It should not be interpreted as an order of clinical relevance
anatomic_site_concept_id	No	integer	This is the site on the body where the feature was found. It maps the ANATOMIC_SITE_SOURCE_VALUE to a Standard Concept in the Spec Anatomic Site domain
alg_system	No	varchar (max)	URI of the algorithm that extracted features, including version information
alg_datetime	No	datetime	The date and time of the algorithm processing

The Image_occurrence table describes imaging events and provides data lineage to the imaging study stored in DICOM format on a medical image storage system, often called a Picture Archive and Communication System (PACS) or Vendor Neutral Archive (VNA). Each row in Image_occurrence represents a collection of images acquired on an imaging modality using a contiguous imaging technique. This is referred to as a DICOM series. Each DICOM series can be an independent modality and acquisition technique grouped within a DICOM study (e.g., PET/CT scan).

The Image_occurrence table has three functions. First, the Image_occurrence table links to DICOM images at a study or a series level. A DICOM study belongs to a single patient, and a patient may have multiple studies. A study is independent of modality whereas a series is dependent on it. A series contains attributes pertaining to frame of reference and equipment, and multiple series can belong to a study. The local_path can be provided in the table to reference the image study or series from imaging storing system. In 2012, DICOM released the DICOMweb standards using web protocols to digitally access DICOM objects (WADO) using RESTful services (RS) and uniform resource identifiers [15] (URI). With the study_UID and series_UID provided in the table, researchers can use the WADO query at the study or series level to retrieve the pixel data and other DICOM attributes. Second, the Image_occurrence table includes series-level parameters. Certain DICOM attributes, such as modality, anatomic site location, and laterality, have been standardized and structured. The anatomic_site_concept_id in the Image_occurrence refers to the body part where the imaging study was performed from the OMOP standard vocabulary. For additional attributes, wadors_uri or local_path can be used to retrieve the required objects. Third, the Image_occurrence table provides provenance for the Image_feature table to identify the images used in creating the features.

Imaging features are comprised of algorithm results executed on the images, image acquisition parameters, and structured radiology reports. The Image_feature table describes the characteristics of the images and their provenance (Table 3). Each row will contain a uniquely identified feature with links to the source imaging as well as the clinical domain table the feature is located in. The image_feature_type_concept_id will describe the method used to create that feature (e.g., machine learning algorithm, DICOM structured report). Image acquisition parameters will also be stored in the clinical domain tables and linked to the source images through the image feature table. This allows the cardinality to include multiple acquisition parameters as well as the benefit of being able to query them with existing tooling in the OHDSI platform.

A set of images may contain multiple imaging findings, each with multiple features. An example would be a chest CT with two nodules where one nodule may be an 8 mm “solid,” and the other may be a 12 mm “part solid.” Each image_feature_concept_id uses the standard vocabulary, often LOINC or RadLex, to identify individual features such as “part solid.” The image_finding_concept_id provides a mechanism to identify concepts for grouped multiple image features such as a “nodule.” The image_finding_id will be a unique key for each imaging finding. The anatomic_site_concept_id in the Image_feature table refers to the specific anatomical location of the feature.

The alg_system field identifies the algorithm information that produces the image finding. API Model serving techniques can be employed to reconstruct the features from computer algorithms [16].

### Integration with Existing OMOP CDM Table

**Fig. 2:**
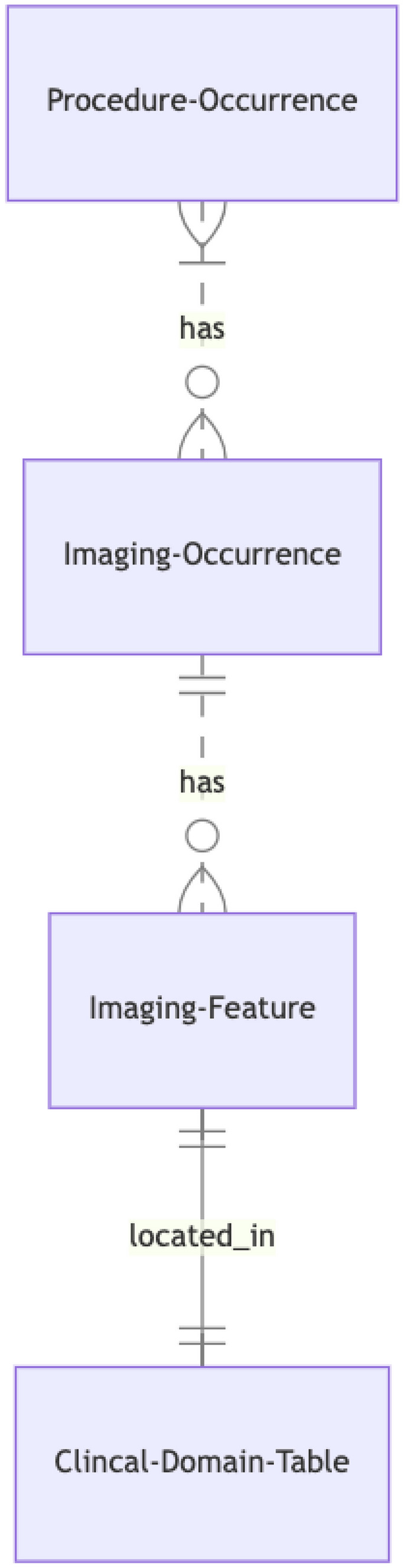
Relationship of new tables to existing OMOP CDM tables

The Image_occurrence table provides information on imaging studies or events. The imaging procedure code for the imaging study is referred to by the procedure_concept_id through the unique identifier procedure_occurrence_id in the Procedure_occurrence table. The Image_occurrence table has a many-to-many relationship with the Procedure_occurrence table, as many CPT codes can be linked to many DICOM series (Fig. 2). For example, a chest X-ray has a one-to-one relationship for linking a single CPT code to the DICOM Series, but chest abdomen pelvis computed tomography (CT) has multiple CPT codes to a single DICOM series. In addition, one magnetic resonance (MR) image procedure will have multiple DICOM series.

The Image_feature table contains information about the features extracted from the findings identified in an image. The Image_feature table has a one-to-many relationship with the Image_occurrence table, as one image series may produce multiple imaging findings (Fig. 2). The Image_feature table contains fields that explain what the imaging findings are, with the values of the findings stored in the clinical domain table. Thus, the Image_feature table has a one-to-one relationship with clinical data tables (e.g., one row in the Image_feature table links to one row in the Measurement table). The image_feature_event_field_concept_id and image_feature_event_id links Image_feature to the relevant clinical data table.

### Conventions for Medical Imaging Tables

The proposed imaging tables enable researchers to define computational phenotypes incorporating imaging features. In our clinical use case, a researcher requires patients with chest computed tomography scans acquired with a slice thickness of 1 mm and 150 kVp for patients who were ultimately diagnosed with lung cancer. The Image_occurrence table holds information about anatomical location, the date of procedures, and the modality. The Image_feature table links to the Measurements table to record findings and features from the image, such as the acquisition parameter slice thickness of 1 mm and a nodule size of 8 mm. Condition_occurrence table includes condition codes generally made at the encounter level linked by the Visit_occurrence table. In our use case, we can search for CT studies performed during an encounter period with a condition occurrence for “lung cancer.” In our model, AI algorithms can even generate classifications, for example, the RadLex code “Lung RAD3,” and link to the Condition_occurrence table through an imaging finding.

The Image_feature table contains two fields that can capture which algorithms were used in the alg_system field and the time of execution in the alg_datetime field. Being able to capture which specific algorithms were used and which are the image feature findings enables the traceability of the AI models and, as such, their reproducibility, contributing to building reproducible AI models in medical imaging [17, 18].

**Fig. 3:**
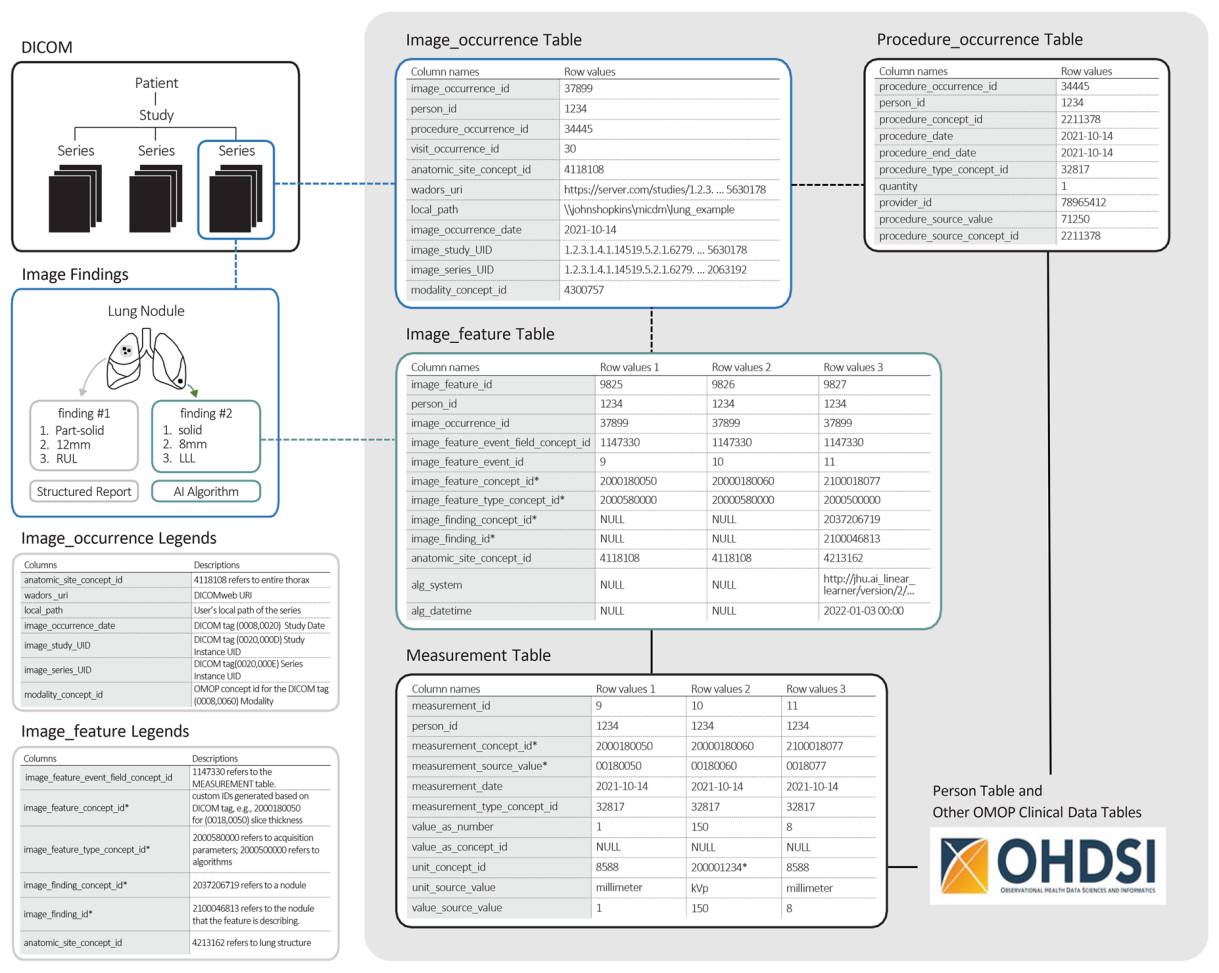
Example of imaging extension tables referencing OMOP clinical data tables for a lung nodule example

Figure 3 demonstrates our use case and how the tables are filled with single imaging occurrence and multiple imaging features. We populated the figure with two acquisition parameters, slice thickness and kVp, and one imaging feature, size of the nodule. The columns with an asterisk indicate custom concept identifier numbers as RadLex and DICOM attributes are not currently in the OMOP vocabulary.

With this extension we can now leverage the Episode and Episode_event tables for tracking imaging findings across successive imaging occurrences. Episode and Episode_event tables were introduced in OMOP CDM 5.4.1 to provide a post coordinated mechanism to link related data in the clinical domain tables [19]. In our example of tracking lung nodules over time to determine a doubling rate, we will create an episode_id for each “nodule” found and link multiple image_finding_ids to the episode_id with the Episode_event table.

**Fig. 4:**
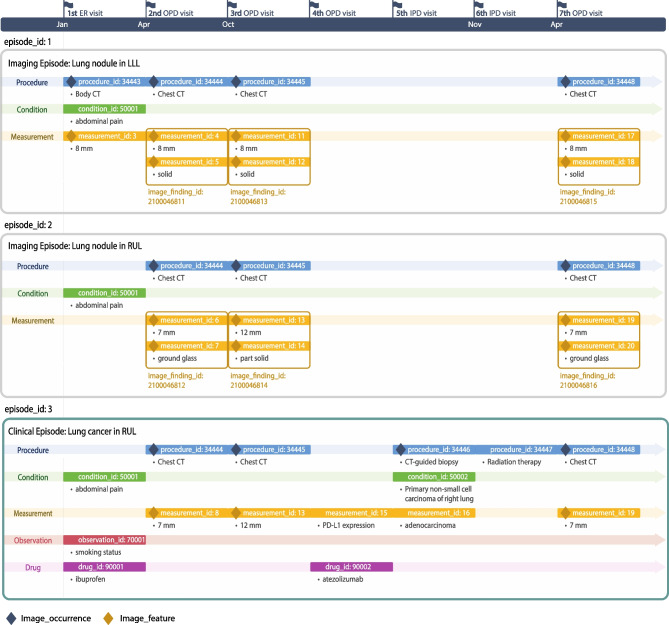
Longitudinal tracking with Episode table and imaging extension tables for lung nodules example

Figure 4 illustrates the utilization of the Episode and Episode_event tables for longitudinal tracking of lung nodules in a patient. The figure contains three blocks, each representing an episode. The first two blocks track imaging episodes of a lung nodule in the left-lower-lobe and the right-upper-lobe, and the last describes the disease progression in the right-upper-lobe. In our example, a patient has a total of seven visits over 16 months, starting from an emergency room visit in January to subsequent visits for diagnosis and treatments. During the initial hospital visit due to abdominal pain, a body CT scan identifies lung nodules. Subsequent outpatient visits focus on monitoring the lung nodules’ progress. During the third visit in October, a chest CT scan revealed growth in the size of the right upper lobe lung nodule. As a result, a CT-guided biopsy is performed during the following visit, leading to a diagnosis of non-small cell carcinoma. Subsequent treatment involves radiation therapy. After a 5-month interval, a follow-up visit is conducted to assess the lesion.

Each episode includes multiple event_ids relating to the episode. The primary key for each data point in the episode, such as procedure_occurrence_id, condition_occurrence_id, and measurement_id, will be recorded in the Episode_event table as event_id to establish connections between the higher-level Episode table and lower-level clinical events. Procedures related to imaging are linked to the Image_occurrence table, and clinical entries from imaging are linked to various clinical tables. These linkages are depicted using diamond-shaped labels. Under the clinical episode in Fig. 4, five measurement data points are linked to the episode, comprised of three imaging features and two lab results. This example demonstrates that the proposed imaging tables can leverage existing episode structure to aggregate events for an imaging episode, and a clinical episode can incorporate imaging signatures along with other clinical data elements.

## Discussion

The study proposes an extension model to the existing Observational Medical Outcomes Partnership Common Data Model (OMOP CDM) to integrate medical imaging data. This extension adds two new tables—Image_occurrence and Image_feature—aligned with existing OMOP CDM conventions and designed to capture detailed information about imaging events and features with their provenance. The tables utilize standard vocabularies, such as SNOMED CT, LOINC, DICOM, and RadLex, for semantic consistency and propose a mechanism for longitudinal tracking of multiple imaging features.

It is important to note that this medical imaging extension focuses on the organization of structured imaging data and features generated by AI models and does not encompass narrative text radiology reports or other unstructured DICOM fields. The radiology reports and other unstructured documents require parsing and additional post-processing to be computationally available, and therefore, these elements should be appropriately organized within the NLP_note table. The exception to this is DICOM “structured reports” (SR objects) which are information objects linked to pixel-based measurements.

The extension establishes relationships with existing clinical data tables and offers the capability to include algorithmic metadata for features extracted through computational methods. Although provenance is provided to record the utilization of AI algorithms in generating image features, the study does not delve into the validation metrics or fields associated with these algorithms. Future studies should consider adding validation guidance and fields for algorithms and inter- and intra-reader variations. Additionally, the current data model lacks provisions for connecting multiple image sets from different studies that may be employed to detect changes across numerous occurrences of an image, such as tracking the progression of a lung nodule over a period. DICOM SR objects support a “Tracking UID” for individual findings over time, which should be incorporated in this MI-CDM as this attribute becomes more widely implemented. Another method for change detection could be creating a new series of images representing the difference of the current image set co-registered with the past image sets.

While the medical imaging extension addresses the research needs of imaging data within the person-centric design of the OMOP CDM, certain limitations must be considered. One limitation is that the proposed model allows granularity only down to the series level, as opposed to the instance level available in the DICOM standards. Researchers can address this in a variety of techniques from (1) including the image instance detection part of the algorithm, (2) create new series with only the key image(s) needed for the algorithm, (3) or further extend the data model to include the image instance IOD. Furthermore, this study assumes that the medical images are in the DICOM format. This is the norm in clinical settings, except for some photographic images (e.g., dermatology). Other imaging formats used in medical imaging research are often transformed from DICOM and often lose considerable metadata in the process. While the examples in this paper are focused on radiology, this model can be extended to other clinical areas which use DICOM, such as cardiology and pathology.

The evolving ACR/RSNA Common Data Elements (CDE) initiative has the goal of creating more consistent and well-defined key-value pairs.

## Conclusion

The extended data model offers a comprehensive and unified approach for conducting imaging research and outcome studies utilizing imaging features. This enables storage and retrieval of medical images and facilitates cross-study comparisons and collaboration across different institutions. Moreover, including imaging features within the OMOP CDM broadens the scope of observational research, allowing for more comprehensive investigations into the associations between imaging findings and various clinical outcomes. The next step in this work is to seek feedback and develop reference implementations to be conducted by the OHDSI Medical Imaging Working Group.

## Data Availability

This work did not use real-world data to conduct the study.
